# Missed losses loom larger than missed gains: Electrodermal reactivity to decision choices and outcomes in a gambling task

**DOI:** 10.3758/s13415-015-0395-y

**Published:** 2015-12-14

**Authors:** Yin Wu, Eric Van Dijk, Mike Aitken, Luke Clark

**Affiliations:** Behavioural and Clinical Neuroscience Institute, Department of Psychology, University of Cambridge, Cambridge, CB2 3EB UK; Department of Social and Organizational Psychology and Leiden Institute for Brain and Cognition, Leiden University, Leiden, The Netherlands; Department of Psychology, Institute of Psychiatry Psychology and Neuroscience, King’s College London, London, UK; Centre for Gambling Research at UBC, Department of Psychology, University of British Columbia, Vancouver, British Columbia Canada

**Keywords:** Prospect theory, Loss aversion, Near-miss, Arousal, Somatic marker hypothesis

## Abstract

Loss aversion is a defining characteristic of prospect theory, whereby responses are stronger to losses than to equivalently sized gains (Kahneman & Tversky *Econometrica, 47*, 263–291, 1979). By monitoring electrodermal activity (EDA) during a gambling task, in this study we examined physiological activity during risky decisions, as well as to both obtained (e.g., gains and losses) and counterfactual (e.g., narrowly missed gains and losses) outcomes. During the bet selection phase, EDA increased linearly with bet size, highlighting the role of somatic signals in decision-making under uncertainty in a task without any learning requirement. Outcome-related EDA scaled with the magnitudes of monetary wins and losses, and losses had a stronger impact on EDA than did equivalently sized wins. Narrowly missed wins (i.e., near-wins) and narrowly missed losses (i.e., near-losses) also evoked EDA responses, and the change of EDA as a function of the size of the missed outcome was modestly greater for near-losses than for near-wins, suggesting that near-losses have more impact on subjective value than do near-wins. Across individuals, the slope for choice-related EDA (as a function of bet size) correlated with the slope for outcome-related EDA as a function of both the obtained and counterfactual outcome magnitudes, and these correlations were stronger for loss and near-loss conditions than for win and near-win conditions. Taken together, these asymmetrical EDA patterns to objective wins and losses, as well as to near-wins and near-losses, provide a psychophysiological instantiation of the value function curve in prospect theory, which is steeper in the negative than in the positive domain.

Within human decision-making, it is well recognized that “losses loom larger than gains,” a phenomenon labeled *loss aversion* (Kahneman & Tversky, 1979). For example, when faced with a mixed gamble with a 50 % chance of winning £150 and a 50 % chance of losing £100, most people refuse this gamble, even though the expected value is clearly positive. Typically, the minimum gain that people need to balance an equal chance of losing money is approximately twice the size of the loss (Kahneman & Tversky, 1979). This asymmetry in the values assigned to gains and losses is represented by the “value function” in prospect theory, in that the curve is shallower for gains and steeper for losses. The overweighting of losses may convey adaptive benefits for survival and reproduction (Kahneman, 2011).

Recent work has linked loss aversion to the brain circuitry involved in emotional processing. In a series of gambles with a 50/50 chance of gaining or losing money, individual estimates of behavioral loss aversion were associated with a neural instantiation of loss aversion in ventral striatum and prefrontal cortex (Tom, Fox, Trepel, & Poldrack, 2007). Brain injury to the amygdala was seen to abolish loss aversion (De Martino, Camerer, & Adolphs, 2010), and using functional magnetic resonance imaging (fMRI) in healthy participants, behavioral loss aversion correlated with amygdala response to losses relative to gains (Sokol-Hessner, Camerer, & Phelps, 2013). In addition to its role in threat detection and negative emotional processing, the amygdala mobilizes the autonomic nervous system, substantiating the view that loss aversion is an emotional process (Sokol-Hessner, Hartley, Hamilton, & Phelps, 2015; Sokol-Hessner et al., 2009; Takahashi et al., 2012; see Phelps, Lempert, & Sokol-Hessner, 2014, for a review). Indeed, propranolol, a norepinephrine beta-blocker that acts primarily to attenuate autonomic arousal, was recently seen to reduce loss aversion (Sokol-Hessner, Lackovic, et al., 2015).

Psychophysiology is a useful tool for characterizing these emotional influences. In the present study, we focused on electrodermal activity (EDA) as a marker of sympathetic nervous system activity, which is known to scale with the dimension of arousal, representing emotional intensity (see Dawson, Schell, & Filion, 2007, for a review). In experiments using the Iowa Gambling Task, EDA in the few seconds before choices (“anticipatory skin conductance responses”) discriminated between advantageous and disadvantageous decisions (Bechara, Damasio, Tranel, & Damasio, 1997). The Iowa Gambling Task conflates responses to risk and to uncertainty with those driving learning of the advantageous strategy, but in a task offering a 50/50 chance of winning or losing money, losses were seen to elicit greater EDA than wins, and this differential arousal correlated with a behavioral index of loss aversion (Sokol-Hessner et al., 2009). In this study, the researchers sought to extend these observations to betting behavior in a task with no overt learning component. Using a wheel-of-fortune game, participants placed a bet on whether the wheel would stop on a winning, losing, or neutral segment. We recorded EDA during both the bet selection and outcome phases. We predicted that selection-related EDA would scale linearly with bet size (see also Studer & Clark, 2011). We further predicted that outcome-related EDA would increase as a function of the magnitudes of wins and losses. On the basis of the phenomenon of loss aversion, we predicted a steeper magnitude function for losses than for wins.

The second objective of our study was to investigate EDA reactivity to salient, nonobtained outcomes. *Counterfactual thinking* refers to the mental process by which people consider salient alternatives to the events that actually happened (Roese, 1997). Upward counterfactual thoughts involve the comparison of the obtained outcome with a more desirable alternative (eliciting regret), whereas downward counterfactual thoughts involve the comparison of the obtained outcome with a less desirable alternative (prompting relief). These counterfactuals have large impacts on emotional responses and behavioral regulation (see Epstude & Roese, 2008, for a review).

Previous research has shown that regret is associated with significant EDA increases (Camille et al., 2004). In a combined fMRI and EDA study using electric shocks as outcomes, Chandrasekhar, Capra, Moore, Noussair, and Berns (2008) reported that regret (e.g., narrowly missing the safe outcome and being shocked) elicited greater EDA than relief (e.g., narrowly missing electric shock and being safe), but the direct contrast of upward and downward counterfactuals in that study was confounded by delivery of the electric shock. The use of a wheel-of-fortune task in the present study enabled near outcomes, in which the spinner stopped just next to either the win (i.e., near-win) or the loss (near-loss) segment (see Fig. 1), to be directly compared while keeping the objective value of the outcome neutral. To index counterfactual thinking, participants provided trial-by-trial ratings of perceived luckiness (Wu, van Dijk, & Clark, 2015). Near-wins have previously been shown to elicit downward counterfactual thoughts and decreased self-perceived luck, whereas near-losses elicited upward counterfactual thoughts and increased self-perceived luck (Wohl & Enzle, 2003; Wu et al., 2015). Near-wins and near-losses also have differential effects on a facial electromyography index of positive affect (Wu et al., 2015). We predicted that EDA would increase for both near-wins and near-losses in this study.

**Fig. 1:**
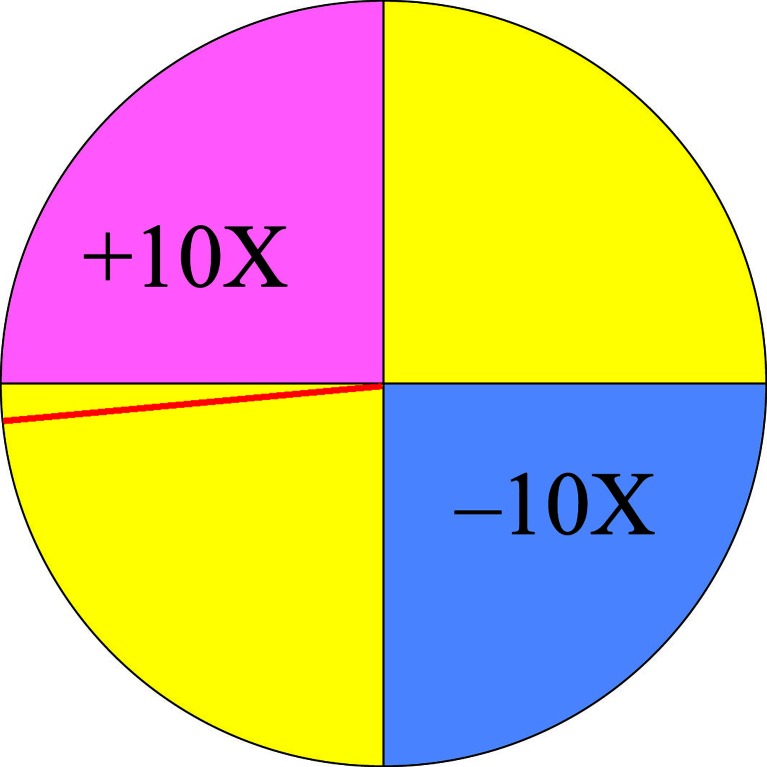
Wheel-of-fortune task. The final position of the red line indicated the outcome—a near-win outcome, in this example

## Method

### Participants

We recruited 51 healthy volunteers (26 men, 25 women; mean age = 24.5 years, *SD* = 4.2, age range = 19–35) from the student population at the University of Cambridge for a study of gambling behavior. We determined this sample size on the basis of previous EDA studies (Clark, Li, et al., 2012; Lang, Greenwald, Bradley, & Hamm, 1993). Our recruitment strategy excluded psychology and economics students. The study was conducted in accordance with the Declaration of Helsinki and was approved by the University of Cambridge Psychology Research Ethics Committee. Written informed consent was obtained from all participants. Volunteers attended an individual testing session and were paid a fixed fee as reimbursement for their time, plus a financial bonus that was proportional to their actual earnings in the gambling task. During their session, participants also completed an emotional reactivity task and a regret task that have been reported elsewhere (Wu & Clark, 2015).

### Wheel-of-fortune task

Participants played a computerized wheel-of-fortune game adapted from Wu et al. (2015; see Fig. 1). The task was programmed in MATLAB using the Psychophysics Toolbox extensions (Brainard, 1997). On each trial, the wheel was divided into four segments: a gain and a loss segment (of different colors) were always separated by two “null outcome” segments. The + or – symbols in each segment indicated the amounts that participants stood to win or lose, and the number (e.g., 10) indicated the size of the win/loss, as a multiplier of the amount that participants bet on each round. For instance, +10X meant that the participant would win 10 times the amount bet, and –10X meant that he or she would lose 10 times the amount bet. In an important modification of our previous task (Wu et al., 2015), rather than depicting the spin by highlighting each segment successively, we used a line vector as the spinner, which allowed the outcomes to fall at varying distances from the boundary to the next segment.

**Fig. 2:**
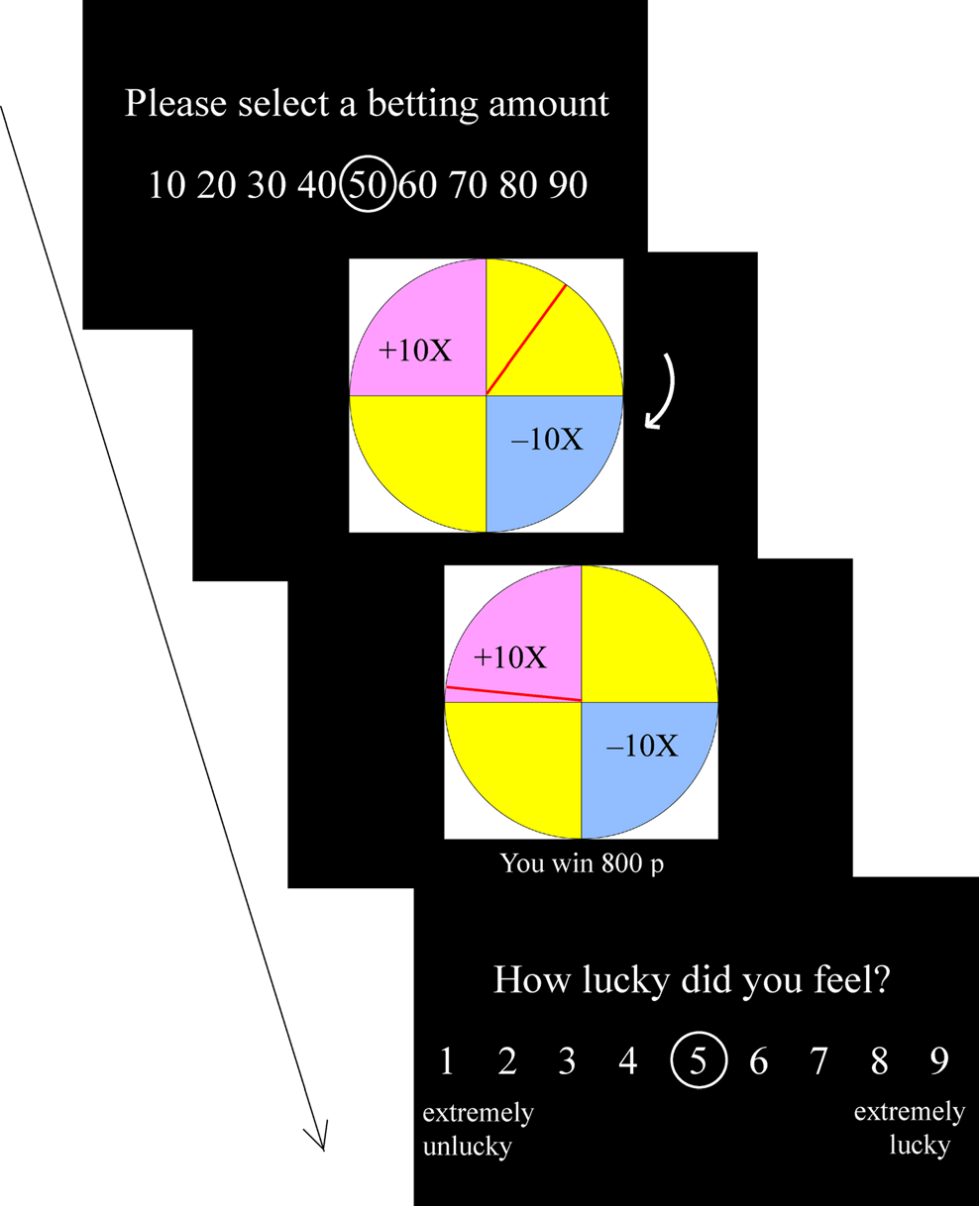
Sequence of events in a single trial. The arrow on the second screen indicates the movement direction of the spinner. This trial displays a win outcome, on which the participant has won 10 times the amount bet

At the beginning of each trial, the participant was asked to choose a bet between £0.10 and £0.90, displayed in £0.10 increments (see Fig. 2). Bets were anchored at £0.50, and the participant adjusted the bet size by moving the circle with the left and right arrow buttons on the keyboard. Following bet selection, the spinner on the wheel spun for an anticipation interval (5.3–6.9 s), during which the spinner decelerated to a standstill. The outcome phase then lasted 1 s, during which the spinner stopped, with accompanying auditory feedback (an applause sound for winning outcomes, a booing sound for losing outcomes, and a thud sound for null outcomes), and the numeric outcome was displayed for 1 s. Following the outcome phase, a luck rating was displayed using a 9-point visual analogue scale (“How lucky did you feel?”), with 1 indicating *extremely unlucky* and 9 indicating *extremely lucky*. The default luck rating was 5, and the participant could adjust the number by moving the circle with the left and right arrow buttons. No time constraints were imposed on bet selection or luck ratings. During a 10-s intertrial interval, only a fixation cross was displayed, to allow for recovery of the physiological signals.

Participants completed 76 trials in total, comprising 19 wins and 19 losses, respectively, in which participants either won or lost 10 times their bet, respectively. Ten near-wins and ten near-losses were delivered, in which participants experienced a null outcome that was close (within 1.8° of the segment boundary) to a major win or loss. To enhance the participant’s impression that the spinner was random, we included 18 filler trials in which the spinner landed near the center of the null segment, close to neither a win nor a loss. We did not include the filler trials in our analysis strategy. The participants were given a funnel debriefing after testing (i.e., starting with broad, open questions about the purpose of the experiment and gradually narrowing down); no participants indicated any suspicions regarding the delivered outcomes.

### Recording equipment

During the gambling task, EDA was recorded via a BIOPAC MP36 unit (BIOPAC Systems Ltd., Goleta, CA, USA), following the previous methods (Clark, Crooks, Clarke, Aitken, & Dunn, 2012; Clark, Li, et al., 2012). Facial muscle reactivity on the zygomaticus and corrugator sites was also collected during the task, but these results are reported elsewhere. The BIOPAC unit, sampling at 1000 Hz, was connected to the stimulus delivery computer and to a second recording computer running AcqKnowledge 4.1 software. The task-related events occurring on the stimulus delivery computer were synchronized to the psychophysiological trace using digital channels. EDA was measured using two grounded Ag–AgCl electrodes (a BIOPAC TSD203 transducer with a GSR100C amplifier module; gain = 5 V, low-pass filter at 1.0 Hz, high-pass filter to block DC), secured on the distal phalange of the index and middle fingers of the nondominant hand. Isotonic paste (BIOPAC Gel101, with a recommended NaCl concentration of 0.05 M) was used as the electrolyte, and participants washed their hands prior to attachment of the electrodes. The EDA signal was transformed into units of microsiemens (*μ*S) using AcqKnowledge. Following attachment of the EDA electrodes, 5 min of resting-state data were acquired, to allow for signal stabilization, prior to commencing the gambling task.

### Data analysis

The data were screened prior to analysis and resampled at 100 Hz. Of the 51 participants, one did not exhibit any phasic changes in EDA and did not show a reliable response to any stimuli, and thus was excluded from the analysis as a nonresponder. The EDA data were logarithmically transformed, given their typical positive skew distribution (Boucsein, 1992), and extracted for the selection phase and the feedback phase separately, using in-house scripts developed in R Studio (R Development Core Team, 2008). The last 2 s of the intertrial interval (ITI) was used as a trial-by-trial baseline to control for typical low-frequency drift in and EDA signal. For each trial, the mean EDA values were extracted in 4 × 2 s bins from the onset of the choice phase and the onset of the outcome phase. An EDA summary measure was calculated as the maximum change in Bins 2–4 (i.e., 2–8 s postchoice or 2–8 s postoutcome) from the baseline value, given the typical time course for EDA changes (Dawson et al., 2007).

We used R and lme4 (Bates, Maechler, & Bolker, 2012) to perform a linear mixed-effects analysis. We used linear mixed-effects modeling via restricted maximum likelihood for all repeated measures analyses so as to reduce information loss when evaluating large, unbalanced data sets after signal standardization (Judd, Westfall, & Kenny, 2012). The participant ID number was included in the model as a random-effect dummy variable. For selection-related EDA, we used the model EDA ~ Bet + (1 | Participant), where Bet was a continuous fixed-effect factor, and Participant was a random-effect factor. For subjective luck ratings and outcome-related EDA, we used the model EDA (or rating) ~ Outcome Magnitude + (1 | Participant), where Outcome Magnitude was a continuous fixed-effect factor. We first assessed the effects of outcome magnitude for objective win and loss outcomes separately, and then compared the slopes for each participant (i.e., the EDA or luck rating, as a function of the outcome magnitude) between the gain and loss conditions. Similarly, for near-win and near-loss outcomes, we tested the effects of the nonobtained outcome magnitude separately for near-wins and near-losses, and then compared the two slopes derived from each participant. Visual inspection of residual plots did not reveal any obvious deviation from homoscedasticity or normality. All *p* values were derived by the lmerTest package (Kuznetsova, Christensen, & Brockhoff, 2012).

## Results

The average bet latency was 2.91 s (*SD* = 2.0), and the average rating latency was 2.44 s (*SD* = 1.75). On average, participants bet 45 British pence (i.e., £0.45, *SD* = 0.18) on each trial. The mean numbers of trials per participant at each level of bet are shown in Table 1. Overall, participants won money on the task (*M* = £9.59, *SD* = 16.43): one-sample *t* test against zero, *t* = 4.03, *df* = 50, *p* < .001. This appeared to be driven by a betting strategy in which participants reduced their bets following wins (*M* = –5.77 p, *SD* = 8.69), *t* = –4.74, *df* = 50, *p* < .001, to a greater extent than the corresponding increase in bets following losses (*M* = 3.40 p, *SD* = 9.76), *t* = 2.49, *df* = 50, *p* = .016. Since the objective probabilities of winning and losing were equal, this asymmetrical gambler’s fallacy led to participants accumulating a profit in the long run.

**Table 1:** Number of trials under each bet size

	10 p	20 p	30 p	40 p	50 p	60 p	70 p	80 p	90 p
Mean	15.41	10.31	8.47	6.08	9.80	4.06	3.76	3.12	14.98
*SD*	16.61	9.63	8.28	7.12	9.98	5.11	4.91	4.16	20.09

### Selection-related EDA

**Fig. 3:**
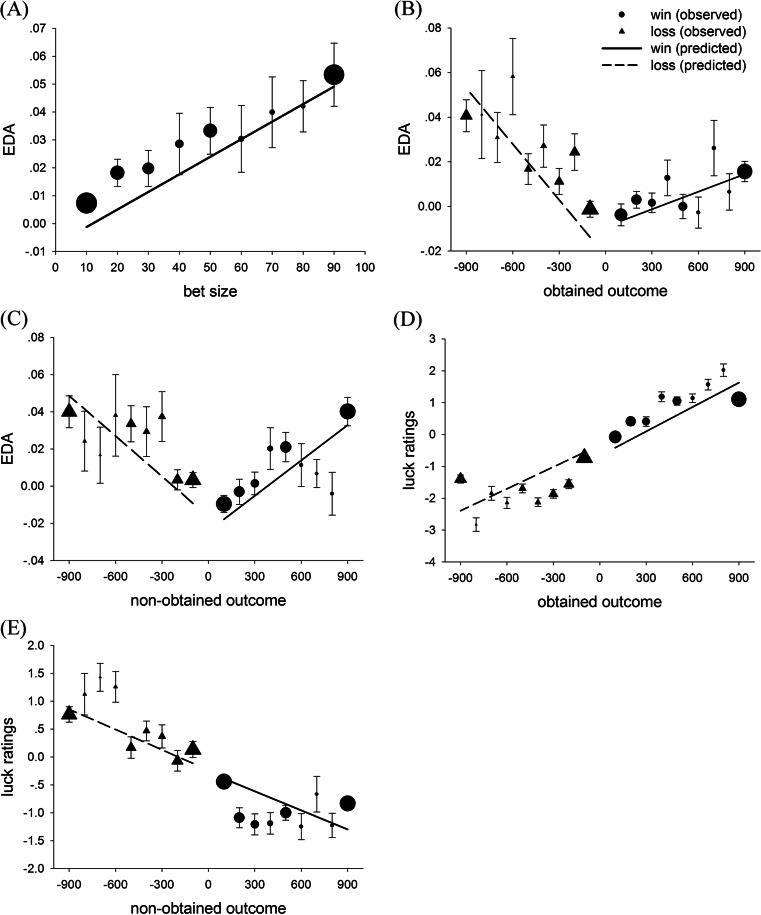
Psychophysiological activity and subjective ratings during the wheel-of-fortune task. **a** Electrodermal activity (EDA) responses to bet size. **b** EDA responses to wins and losses. **c** EDA responses to near-wins and near-losses. **d** Luck ratings for wins and losses. **e** Luck ratings for near-wins and near-losses. For all graphs, error bars represent standard errors of the means of the observed data, and the fitted lines are derived from regression models. The size of each data point is proportional to the number of observations within each panel

When fitting selection-related EDA, we found a significant linear relationship between bet size and EDA, *b* = 6.30 × 10^–4^, *SE* = 9.15 × 10^–5^, *t* = 6.88, *p* < .001, such that higher bets were associated greater EDA (see Fig. 3a).

### Outcome-related EDA

#### Wins versus losses

When we fitted outcome-related EDA, a significant linear relationship emerged between the size of the win and EDA, *b* = 2.625 × 10^–5^, *SE* = 7.474 × 10^–6^, *t* = 3.15, *p* < .001, and between the size of the loss and EDA, *b* = 8.35 × 10^–5^, *SE* = 1.01 × 10^–5^, *t* = 8.27, *p* < .001, with larger monetary outcomes being associated with a greater EDA increase. In a direct test, the EDA slope (as a function of outcome magnitude) was significantly steeper for losses than for wins, *t* = 5.760, *p* < .001 (see Fig. 3b).

#### Near-wins versus near-losses

We also found a significant linear relationship between the magnitude of the nonobtained win (i.e., near-win) and EDA, *b* = 6.34 × 10^–5^, *SE* = 9.79 × 10^–6^, *t* = 6.48, *p* < .001, and between the magnitude of the nonobtained loss (i.e., near-loss) and EDA, *b* = 7.23 × 10^–5^, *SE* = 1.21 × 10^–5^, *t* = 6.00, *p* < .001, such that nearly missing higher-magnitude outcomes (either gains or losses) elicited stronger EDA.^1^ Similar to the effect for the objective outcomes, the slope of EDA sensitivity was steeper for near-losses than for near-wins, *t* = 2.46, *p* = .017 (see Fig. 3c). Although the trend lines for near-wins and near-losses appear to be of similar steepness in Fig. 3c, we note that the lower EDA responses in the near-win upper range (600–800) are for trials with a limited number of observations (indicated by the relative sizes of the data points).

### Relationships between selection-related and outcome-related EDA

The slope of selection-related EDA correlated significantly with the slopes of EDA for both wins, *r* = .55, *p* < .001, and losses, *r* = .76, *p* < .001. A Williams test indicated that the slope of selection-related EDA was correlated to a greater degree with the slope of EDA for losses than with the slope of EDA for gains, *t* = 2.53, *df* = 47, *p* = .015. The slope of selection-related EDA also significantly correlated with the slopes of EDA for both nonobtained wins (near-wins), *r* = .48, *p* < .001, and nonobtained losses (near-losses), *r* = .83, *p* < .001. Again, the slope of selection-related EDA showed a stronger correlation with the slope of EDA for near-losses than with the slope of EDA for near-wins, Williams test *t* = 4.15, *df* = 47, *p* < .001.

### Luck ratings

#### Wins versus losses

A significant linear relationship was apparent between the size of a win and the subsequent luck ratings, *b* = 2.56 × 10^–3^, *SE* = 1.64 × 10^–4^, *t* = 15.66, *p* < .001, such that larger gains were perceived as being luckier. There was also a significant linear relationship between the size of a loss and luck ratings, *b* = 2.29 × 10^–3^, *SE* = 1.55 × 10^–4^, *t* = 14.79, *p* < .001, such that larger losses were perceived as being unluckier. A direct test of the difference between the two slopes for luck ratings as a function of win and loss magnitude was not significant, *t* = 0.85, *p* > .250 (see Fig. 3d).

#### Near-wins versus near-losses

We found a significant linear relationship between the size of a nonobtained win (i.e., near-win) and luck ratings, *b* = –1.34 × 10^–3^, *SE* = 1.79 × 10^–4^, *t* = –6.36, *p* < .001, with larger nonobtained wins being perceived as unluckier. We also discovered a significant linear relationship between the size of a nonobtained loss (i.e., near-loss) and luck ratings, *b* = –1.21 × 10^–3^, *SE* = 2.25 × 10^–4^, *t* = –5.38, *p* < .001. No significant difference was apparent between the slope of luck ratings as a function of the magnitude of near-wins and the slope of luck ratings as a function of the magnitude of near-losses, *t* = 0.39, *p* > .250 (see Fig. 3e).

## Discussion

Using a gambling task, we first observed that outcome-related EDA varied positively with the magnitude of the obtained outcomes, in response to both winning and losing, with the strongest responses occurring for large wins or losses. This is in keeping with a substantial literature showing that EDA is a sensitive marker of emotional *intensity* (Dawson et al., 2007). For example, EDA increased linearly with self-reported arousal in response to emotional images from the International Affective Picture System (Lang, Greenwald, Bradley, & Hamm, 1993). However, in that study the slope of EDA as a function of the size of the obtained outcomes was reliably steeper for losses than for gains, consistent with a previous observation that losses elicit stronger EDA than equivalent-sized gains (Sokol-Hessner et al., 2009).

Importantly, EDA also varied positively with the magnitude of counterfactual outcomes, for both missed gains (i.e., near-wins) and missed losses (i.e., near-losses), such that larger missed outcomes evoked stronger EDA. Past work has shown that near-wins delivered on slot machines (e.g., two cherries on the pay line, with a third cherry falling just short) were associated with EDA increases (Clark, Crooks, Clarke, Aitken, & Dunn, 2012; Clark et al., 2013; Dixon et al., 2011). The present data extend these earlier observations in at least two important ways. First, the effect is not restricted to near-wins, but is also seen following near-losses (and is in fact stronger for near-losses). Second, the processing of near outcomes, in terms of both EDA and luck ratings, scales positively with the *magnitude* of the outcome that was missed. This observation strengthens an interpretation of these narrowly missed outcomes in terms of counterfactual thinking, whereby near-wins induce upward counterfactual thoughts and elicit regret, whereas near-losses induce downward counterfactuals and elicit relief (Wu et al., 2015; Zhang & Covey, 2014).

In addition, the slope of EDA as a function of the magnitude of narrowly missed losses (i.e., near-losses) was steeper than the slope for narrowly missed wins (i.e., near-wins), mimicking the EDA finding that losses loom larger than gains (Sokol-Hessner et al., 2009). Although this is a smaller effect than the difference for obtained losses and gains (see Fig. 3c), it is statistically reliable, and the trend line for near-wins in Fig. 3c is likely biased by the smaller number of observations in the upper range. According to the value function curve, near-wins are represented in the shallow part of the gain curve, and near-losses are represented in the steep part of the loss curve. Because the value function curve is asymmetrical, such that the loss curve is much steeper than the gain section, the elimination of a loss (i.e., a near-loss) has stronger impact on subjective value than does the elimination of a gain (i.e., a near-win), and this is indexed by steeper EDA responses to near-losses than to near-wins.

In describing the value function, previous research in behavioral economics has relied heavily on the use of anecdotal scenarios to elicit subjective responses to loss and nongain events (Kahneman, 2011; Kahneman, Knetsch, & Thaler, 1986). In a classic “framing” study, the loss could be formulated as a price increase or a cut in wages, as compared to the nongain, formulated as the cancellation of a former discount or wage increase (Kahneman et al., 1986). The observation that people respond more strongly to near-losses than to objectively equivalent near-gains has been interpreted in terms of the (anticipated) loss accessing the steeper convex region of the value function, whereas the (anticipated) gain is evaluated against the shallower concave region. We utilized EDA as an objective measure of emotional intensity. An important advantage of using a multishot laboratory task is that we were able to deliver near-wins and near-losses that were *objectively identical* neutral outcomes. EDA further shows high sensitivity to the magnitudes of both obtained and nonobtained outcomes, allowing a fuller characterization of the shape of the value function for this psychophysiological index.

Near-wins decreased and near-losses increased self-perceived luck, consistent with our previous observations (Wu et al., 2015). Self-perceived luck varied linearly as a function of the magnitude of the counterfactual outcome for both near-wins and near-losses, with the largest counterfactual outcome being perceived as most unlucky (or lucky). However, the response patterns for the luck ratings did not differ reliably across near-wins and near-losses, or between objective wins and losses. It is likely that luck ratings emphasize the perception of chance (Teigen, 1995), as opposed to hedonic or motivational aspects of outcome processing (Clark, Lawrence, Astley-Jones, & Gray, 2009), and future research will be needed to corroborate these asymmetries with a broader range of affective ratings.

Previous research has suggested that sounds can increase physiological arousal and play an important role in the maintenance and reinforcement of gambling behavior (Dixon et al., 2014). Here, losing outcomes were accompanied by a “booing” sound, whereas wins were paired with an “applause” sound. It is conceivable that differences in the emotional potencies or saliences of these audio clips could have contributed to the asymmetrical pattern observed for obtained losses and wins, but critically, this cannot underlie the correlations between the EDA responses and the *magnitude* of wins and losses. Furthermore, *near*-wins and *near*-losses were both accompanied by an identical “thud” sound, and thus the asymmetrical EDA response pattern cannot be a sensory effect, but must be attributable to the subjective appraisal of these outcomes.

Selection-related EDA varied positively with the bet size, with the greatest EDA to large bets. Early work on “anticipatory skin conduction responses” described somatic markers during risky as compared to safe decisions in an environment in which participants were required to learn the gain and loss contingencies of different decks (Bechara et al., 1997). Subsequent work has shown physiological signaling in a task without any overt learning requirement, with high bets being accompanied by greater EDA in a binary manner (Studer & Clark, 2011). A recent study showed that EDA is sensitive to explicit risk, defined as the variance across possible outcomes within a gambling option, with a high-risk option eliciting large EDA (Holper, Wolf, & Tobler, 2014). The present study has more fully revealed the exquisite sensitivity of EDA reactivity as a linear function of bet size. In our task, the expected values were neutral across all trials, and the magnitudes of potential wins and losses were explicitly presented; therefore, there was no optimal strategy to be learned or any requirement for sampling or exploration. This finding adds to research on the somatic marker hypothesis by using a decision-making task without any learning or ambiguous context (Crone, Somsen, Van Beek, & Van Der Molen, 2004; Damasio, 2008).

Across individuals, the slope of selection-related EDA correlated with the slopes of EDA for both factual outcomes (wins and losses) and counterfactual outcomes (near-wins and near-losses). This correlational result is consistent with a key tenet of the somatic marker hypothesis: that psychophysiological responses to rewarded or punished outcomes are reactivated during subsequent decisional processing (“deliberation”). As a more unexpected result, the slope of selection-related EDA was correlated more strongly (according to a formal Williams test) with the EDA slope for losses than with the EDA slope for gains. Similarly, the slope of selection-related EDA was correlated more strongly with the EDA slope for near-losses than with that for near-wins. This represents a further instance of “losses looming larger,” and more generally it implicates an updating process through which choice outcomes shape subsequent risk-sensitive decisions, disproportionately weighing negative outcomes over positive outcomes. We interpret this as evidence that the physiological states associated with loss aversion feed forward to bias subsequent risky betting decisions.

Some limitations of the study should be noted. Changes in EDA are sluggish, and our design did not vary (jitter) the interval between the gambling outcomes and the subsequent rating, raising the possibility that the EDA responses time-locked to outcome delivery may have been influenced by the subsequent luck ratings. Indeed, in past work, introspective emotional ratings have been found to amplify stimulus-related physiological responses (Hutcherson et al., 2005), but this is unlikely to account for the differential responses to gain- and loss-related outcomes in our results. Primarily, the EDA slopes for losses and near-losses were reliably steeper than the corresponding slopes for wins and near-wins, whereas such differences were not observed on the luck ratings. As such, we infer that EDA responses reflect outcome appraisal processes more closely than do luck ratings. In addition, the significant correlations between bet- and outcome-related EDA point to an updating mechanism rather than an introspective process. Future decision-making research could benefit by using measurements with better temporal resolution—for example, pupil dilation (Lempert, Glimcher, & Phelps, 2015). For instance, recent studies have shown that pupil dilation is associated with a shift between exploitation and exploration in a reinforcement-learning task (Jepma & Nieuwenhuis, 2011), as well as with risk prediction error in a gambling task (Preuschoff, ’t Hart, & Einhäuser, 2011).

Taken together, these asymmetrical responses in EDA to objective wins and losses, as well as to counterfactual outcomes (near-wins and near-losses), provide a psychophysiological instantiation for the value function in prospect theory, which is steeper in the negative than in the positive domain. The further sensitivity of EDA to betting behavior at the point of decision, along with associations between decision- and outcome-related activity, help to integrate this concept from behavioral economics with the notion of somatic markers from decision neuroscience.

## Footnotes

1 The average EDA response when the spinner was in the middle of the null segment was 0.002 (*SD* = 0.056), collapsing together the two events of the spinner leaving a win or a loss segment.
